# SMG6 Cleavage Generates Metastable Decay Intermediates from Nonsense-Containing β-Globin mRNA

**DOI:** 10.1371/journal.pone.0074791

**Published:** 2013-09-25

**Authors:** Roshan Mascarenhas, Julie A. Dougherty, Daniel R. Schoenberg

**Affiliations:** 1 Center for RNA Biology, The Ohio State University, Columbus, Ohio, United States of America; 2 Department of Molecular and Cellular Biochemistry, The Ohio State University, Columbus, Ohio, United States of America; 3 Biomedical Sciences Graduate Program, The Ohio State University, Columbus, Ohio, United States of America; Korea University, Republic of Korea

## Abstract

mRNAs targeted by endonuclease decay generally disappear without detectable decay intermediates. The exception to this is nonsense-containing human β-globin mRNA, where the destabilization of full-length mRNA is accompanied by the cytoplasmic accumulation of 5′-truncated transcripts in erythroid cells of transgenic mice and in transfected erythroid cell lines. The relationship of the shortened RNAs to the decay process was characterized using an inducible erythroid cell system and an assay for quantifying full-length mRNA and a truncated RNA missing 169 nucleotides from the 5′ end. In cells knocked down for Upf1 a reciprocal increase in full-length and decrease in shortened RNA confirmed the role of NMD in this process. Kinetic analysis demonstrated that the 5′-truncated RNAs are metastable intermediates generated during the decay process. SMG6 previously was identified as an endonuclease involved in NMD. Consistent with involvement of SMG6 in the decay process full-length nonsense-containing β-globin mRNA was increased and the Δ169 decay intermediate was decreased in cells knocked down for SMG6. This was reversed by complementation with siRNA-resistant SMG6, but not by SMG6 with inactivating PIN domain mutations. Importantly, none of these altered the phosphorylation state of Upf1. These data provide the first proof for accumulation of stable NMD products by SMG6 endonuclease cleavage.

## Introduction

Endonuclease decay was thought to play a minor role in mRNA turnover before results from deep sequencing showed widespread evidence for endonuclease cleavage throughout the mammalian mRNA transcriptome [1], [2]. Despite this relatively little is known about the enzymes that generate these cleavages, and only a few bona fide mRNA endoribonucleases have been identified and characterized [3]. A major complication to the study of endonuclease-mediated mRNA decay is the rapidity with which cleavage products are cleared by 5′-3′ and 3′-5′ exonucleases [3]. For the most part decay intermediates are only detected by knocking down Xrn1 to stabilize the downstream fragment [4] or by PCR amplification after ligating a primer to the newly formed 3′ ends of cleavage products [5]. A possible exception to this is the decay of nonsense-containing β-globin mRNA in erythroid cells.

In 1989 Lim and Maquat [6] showed that 5′-truncated forms of human β-globin mRNA accumulate in erythroid cells of mice that are transgenic for several nonsense containing alleles. The same 5′-truncated RNAs accumulate in murine erythroleukemia cells that are stably transfected with wild type and nonsense-containing human β-globin genes [7], [8]. We showed previously that these shortened RNAs were generated by endonuclease cleavage [7], but because they were only seen in erythroid cells it was unclear if these are intermediates in the decay process or the products of a cell type-specific processing that is unique to β-globin mRNA in its native cell environment. Complicating matters further the same 5′-truncated RNAs were also seen in cells expressing wild type β-globin mRNA, albeit at a much lower level [7], and their quantity is increased by coexpressing *Xenopus* PMR1 in these cells [8]. This was originally interpreted as evidence that erythroid cells employ a PMR1-like endonuclease to degrade β-globin mRNA, but that finding preceded the identification of SMG6 as an endonuclease that catalyzes the degradation of nonsense-containing mRNA [9], [10].

Progress in studying endonuclease decay has been limited by the challenges inherent in quantifying short-lived decay intermediates. Thus, if the shortened forms of β-globin mRNA are indeed decay intermediates we could take advantage of their appearance to address several questions about the decay process. To address this we developed an inducible line of erythroid cells which were used to monitor the cytoplasmic appearance of full-length normal (WT-hβG) and PTC-containing (PTC-hβG) human β-globin mRNA after treating cells with doxycycline to induce transcription of their respective genes. Changes in full-length mRNA and one of the 5′-truncated RNAs were determined using a modification of Molecular Beacon Rapid Amplification of cDNA Ends (MBRACE) [11], a qRT-PCR-based assay for quantifying products after ligation of a common primer to uncapped 5′ ends.

## Materials and Methods

### Plasmid Constructs

A wild type (WT) human β-globin gene and a gene with a nonsense codon at position 60/61 (PTC60/61, [7]) were cloned into a modified form of pcDNA3 (pcDNA3/TO) with a tetracycline operator element upstream of the multiple cloning site. Destabilized forms of each of these were generated by site-directed mutagenesis of the nucleolin/α-CP binding site (H124 mutation) [12]. Plasmids expressing wild type and PTC-containing TCRβ mRNA (pAc/IF-TCRβ) [13] were provided by Miles Wilkinson. pcDNA3-HA-SMG6 and SMG6-m4 provided by Oliver Mühlemann were used for complementation experiments. All of the primers described here and below are listed in Table S1.

### Cell Culture

Murine erythroleukemia (MEL) cells that were stably transfected with wild type (Norm2) or nonsense-containing (Thal10) β-globin genes were described previously [7]. Thal10 cells express a form of β-globin mRNA with a single base deletion that results in a nonsense codon at position 60/61 in the mature transcript. Norm2 and Thal10 cells were cultured in Minimal Essential Medium alpha (alpha MEM) supplemented with 10% FBS, and β-globin gene transcription was induced by treating for 48 hr with 1.5% DMSO. K562 human erythroleukemia cells were obtained from the American Type Culture Collection and cultured in the same medium as Norm2 and Thal10 cells. A line of tetracycline inducible K562 cells was generated by transduction with a tetracycline repressor-expressing lentivirus (pLenti6/TR, Invitrogen). Transduction was performed by adding viral supernatant to cells in a 24-well culture plate and centrifuging at 300×g for 2 hr, 25°C using a JS5.3 rotor in a Beckman Avanti J-20 XPI centrifuge. 100 individual colonies were selected by growth for 14 days in medium containing 10 µg/ml blasticidin (Invitrogen) and tested for tetracycline-regulated inducibility by electroporation with pcDNA4/TO/LacZ and with inducible β-globin plasmids and the one showing highest inducibility and tightest regulation was selected for use in this study. Electroporation was performed using a Neon® (Invitrogen) electroporator. 1×10^6^ cells were centrifuged at 100×g for 4 min, the cell pellet was washed with PBS and resuspended in 100 µl of buffer ‘T’ as per manufacturer’s protocol. 3 µg of plasmid DNA was added and electroporation was performed with a 100 µl tip using 2×20 millisecond pulses of 1300 volts.

### siRNA Knockdowns

**Figure 1:**
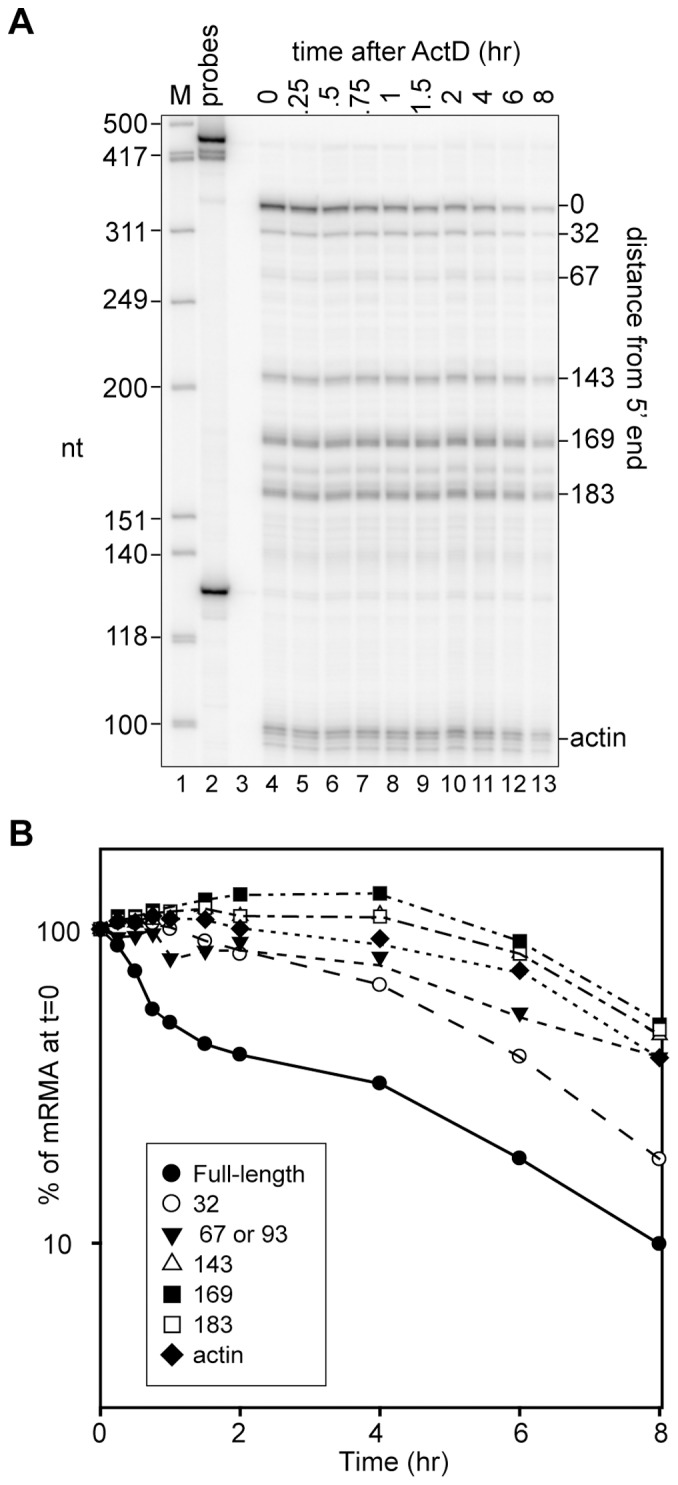
Prolonged cytoplasmic residence of 5′-truncated forms of PTC-hβG mRNA. **A.** Actinomycin D (6 µg/ml) was added to DMSO-induced Thal10 cells at time 0 and cytoplasmic RNA recovered at the indicated times was assayed by S1 nuclease protection using a probe for the first 354 nt of β-globin mRNA. Hinf I restriction fragments of φX174 DNA were used as size markers (M, lane 1) and the location of each of the products relative to the 5′ end of β-globin mRNA is indicated on the right side of the autoradiogram. **B.** The amount of each species was quantified by phosphorimager analysis and plotted on the right as a function of time after addition of Actinomycin D.

For the experiments in Figs. 3 and 5, 1×10^6^ tet-inducible K562 cells electroporated with plasmids expressing inducible forms of β-globin mRNA (WT or PTC60/61) or constitutively expressed forms of T cell receptor (TCR) β (WT or PTC) plasmids were cultured for 16 hr in antibiotic-free medium. The recovered cells were resuspended in 750 µl Accell siRNA delivery media (Thermo, Cat# B-005000). 100 µM solutions of control (Thermo, D-001950-01), Upf1 (Thermo, E-011763-00-0005) or SMG6 (Thermo, E-017845-00-0005) SMARTpool siRNAs were prepared in 1× siRNA buffer (Cat# B-002000-UB-100). 7.5 µl of the 100 µM siRNA solution was added to cells in siRNA delivery media and 100 µl aliquots were placed into individual wells in a 96-well plate. The next day cells were transferred to individual wells of a 24-well plate, and β-globin was induced 48 hr after knockdown by treating for 6 hr with 1 µg/ml doxycycline. A SMG6 siRNA (5′ GCUGCAGGUUACUUACAAG 3′ with 3′ UU overhangs) was generated that corresponds to the shRNA target in [10]. In the experiment in Figure 6 2×10^6^ tetracycline-inducible K562 cells were electroporated with 10 nM of control or SMG6 siRNA in 100 µl of buffer ‘R’. The same cells were electroporated 24 hr later with 10 nM of corresponding siRNA, 20 µg of WT- or PTC-hβG-expressing plasmid, and 20 µg of empty vector, SMG6-expressing plasmid pcDNA3-HA-SMG6 or SMG6 plasmid with the 3 catalytic aspartate residues in the PIN domain changed to asparagine (SMG6-m4, [10]). β-globin gene transcription was induced 16 hr later by adding 1 µg/ml doxycycline to the medium and cytoplasmic RNA was isolated 6 hr after induction. Western blotting was used to determine the effectiveness of knockdown, modified MBRACE assay was used to monitor full-length and Δ169 RNA, and TCRβ mRNA was assayed by qRT- PCR.

### Isolation of Cytoplasmic RNA and Protein

Cell were harvested by centrifugation and washed twice with ice cold PBS. Norm2 and Thal10 cells were resuspended in lysis buffer containing 0.1 M NaCl, 10 mM Tris-HCl pH 8.0, 2 mM EDTA, 1% NP-40, 1% 2-mercaptoethanol, 1 mM dithiothreitol, and 80 units/ml RNaseOUT. K562 cells were resuspended in modified lysis buffer containing 0.15 M NaCl, 50 mM Tris-HCl pH 7.5, 10 mM KCL, 10 mM MgCl_2_, 0.2% NP-40, 2 mM dithiothreitol and 80 units/ml RNaseOUT. These were placed on ice for 5 min, the tubes were gently flicked and incubated on ice for an additional 5 min. The lysates were centrifuged at 5000×*g* at 4°C for 10 min to pellet the nuclei, supernatant (cytoplasmic) fractions were transferred to chilled microcentrifuge tubes and RNA was extracted using Trizol Reagent (Life Technologies) as recommended by the manufacturer’s protocol. Cell lysate was used directly for protein quantification and visualization by western blot analysis.

### Antibodies

Affinity-purified rabbit polyclonal antibody to the tetracycline repressor protein was obtained from MoBiTec. Rabbit polyclonal antibodies to hUpf1 and hSMG6 were provided by Jens Lykke-Andersen, and rabbit polyclonal anti-phosphoUpf1 was obtained from Millipore, Inc. Antibody to ribosomal protein S6, horseradish peroxidase (HRP)-coupled goat anti-rabbit IgG and goat anti-mouse IgG were obtained from Santa Cruz Biotechnology.

### Western Blot Analysis

Samples were denatured in 2× Laemmli sample buffer (Bio-Rad Laboratories) with β-mercaptoethanol. 10 µg of cytoplasmic protein was separated on 10% Mini-PROTEAN® TGX™ precast gels (Bio-Rad Laboratories) and transferred onto Immobilon®-P PVDF membrane (EMD Millipore). Membranes were blocked with 5% nonfat dry milk in Tris-buffered saline containing 0.05% Tween-20 (TBS-T), incubated with primary antibody (1∶1000 dilution) in the same solution, washed with TBS-T, then incubated with horseradish peroxidase-coupled secondary antibody (1∶10000 dilution). For experiments with anti-phospho-Upf1 antibody blots were blocked with 1× TBS +3% BSA for 2 hr at 25°C. They were incubated overnight at 4°C in the same solution with a 1∶250 dilution of anti-phospho-Upf1 antibody and secondary antibody incubation was performed in 1× TBS +3% BSA. Membranes were developed with ECL-plus or for phospho-Upf1 Western blotting a 1∶1 mixture of ECL-prime solutions A and B, and visualized on X-ray film (GeneMate).

### S1 Nuclease Protection Assay

S1 nuclease protection assay was performed as described in [14]. RNA was dissolved in S1 hybridization buffer (80% formamide, 40 mM PIPES, pH6.4, 0.4 M NaCl, 1 mM EDTA) and incubated overnight at 52°C with 3–5×10^4^ dpm of [^32^P]-end-labeled antisense DNA probe. The antisense probe was obtained by asymmetric PCR using the human β-globin cDNA plasmid pSPkβC or the mouse β-actin cDNA plasmid pBSβactin as templates. T4 polynucleotide kinase was used to label the 5′ end of the antisense β-globin primer HBB-AS: beginning at position 250, primer 1249: beginning at position 346 and the antisense β-actin primer YO-41 with γ-[^32^P] ATP (3,000 Ci/mmol). The unlabeled β-globin sense primer (HBB-S) and β-actin sense primer (ACT-S) correspond to sequences just upstream of the β-globin or β-actin cDNA in the expression plasmid. The [^32^P] labeled probe generated by this reaction was purified by electrophoresis on a 6% polyacrylamide/urea gel. After hybridization, S1 nuclease solution (0.28 M NaCl, 0.05 M sodium acetate, pH 5.2, 4.5 mM ZnSO4, 20 µg/ml sheared salmon sperm DNA, 100 units S1 nuclease, Invitrogen) was added to each reaction and the mixture was incubated at 28°C for 2 hr. Samples were precipitated with ethanol and electrophoresed on denaturing 6% polyacrylamide/urea gels. Protected fragments were visualized and quantified by PhosphorImager.

### MBRACE, Modified MBRACE and qRT-PCR Assays

1 µg of DNase I-treated cytoplasmic RNA was incubated with 3 units of tobacco acid pyrophosphatase (TAP, Epicentre) at 37°C for 2 hr in 1× TAP buffer. An RNA adapter (RNA ADP) was ligated to this using T4 RNA ligase I (New England Biolabs) in T4 RNA ligase reaction buffer supplemented with 1 mM ATP at 37°C for 2 hr. cDNA was prepared using SuperScript® III first-strand synthesis system (Life Technologies) and the product was purified using QIAquick® PCR purification columns (Qiagen). Approximately 20 ng of cDNA was used per qPCR reaction containing 500 nM forward (MBRACE-F) and reverse primers (HBB-FL-R1, HBB-Δ169-R1), 250 nM molecular beacon (HBB-FL-MB, HBB-Δ169-MB) in 1× PerfeCTa qPCR FASTmix (Quanta Biosciences). PCR was performed using an Eco Real-Time PCR system (Illumina®) and the following thermal profile: 95°C for 3 min, [95°C 10 sec, 61°C 30 sec with signal detection, 72°C 15 sec] for 40 cycles. Ct baseline and threshold were automatically determined by Eco software. Molecular beacons were designed to the junction sequence between the RNA adapter and full-length or Δ169 β-globin mRNA. HEX-BHQ1 and 6FAM-Dabcyl fluorophore-quencher pairs were used to label the full-length and decay product beacons respectively. HPLC purified beacons were resuspended in 1× TE (10 mM Tris-HCl pH 8.0, 1 mM EDTA) buffer and stored in aliquots at −80°C. Modified MBRACE assay used primers designed to the junction between the ligated adapter RNA and full-length (HBB-JS-F) and Δ169 (HBB-Δ169 -JS-F) RNA. β-globin specific primers (HBB-FL-R1, HBB-Δ169-R1 in MEL, HBB-FL-R2, HBB-Δ169-R2 in K562) were designed to generate similar length products that could be verified by gel electrophoresis. The locations of these primers on hβG mRNA are shown in Figure S1. Reaction conditions to detect full-length β-globin and its decay product were as follows: 20 ng cDNA, 375 nM junction-specific and reverse primers in 1× SYBR Green PCR Master Mix (Applied Biosystems/Life Technologies) in 10 µl reaction volume. The control mRNA TCRβ (TCRβ-F, TCRβ-R), GFP (emGFP-F, emGFP-R), and β-actin (MA-F, MA-R) levels were also quantified by similar method using indicated primers. qPCR was performed on the Eco Real Time PCR system with thermal profiles for all SYBR Green reactions as follows: 95°C for 10 min, [95°C for 10 sec, 60°C 30 sec with signal detection] for 40 cycles followed by a melt curve. ROX present in master mix acted as well-to-well normalization control. The Eco software automatically determined Ct baseline and threshold. To determine the fold change in transfection experiments, β-globin mRNA levels were normalized to the levels of co-transfection control GFP mRNA. In MEL cells β-globin mRNA levels were normalized to β-actin mRNA.

### Data Analysis

Experiments were conducted in biological triplicate and qPCR reactions were performed in sample triplicate. Ct values for each replicate were averaged, ΔCt was calculated by subtracting the control (β-actin or GFP) Ct from the sample Ct. ΔCt values were averaged over the replicates and the average ΔCt for WT β-globin acted as the reference value. The ΔΔCt value was calculated by subtracting the ΔCt of WT from the sample ΔCt value. Fold expression was calculated using the 2∧-ΔCt method [15]. Statistical analysis was performed using JMP9 (SAS, North Carolina) software. Data distribution and variance determined the appropriate method of analysis, all of which were performed 2-tailed with an α = 0.05. Graphs were generated using GraphPad Prism 5 (GraphPad Software, Inc.) and bars represent standard deviation.

## Results

### Actinomycin D Chase Shows Prolonged Presence of 5′ Truncated RNAs in the Erythroid Cell Cytoplasm

Our previous work used murine erythroleukemia cells that were stably transfected with WT-hβG (Norm2) or PTC-hβG (Thal10) transgenes to study β-globin mRNA decay [7], [8]. Using Actinomycin D and Northern blotting we showed that full-length wild type mRNA has a half-life of 12 hr in Norm2 cells and PTC-containing mRNA decays with biphasic kinetics and a half-life of 100 min [8]. Neither of the earlier studies looked at the fate of the 5′-truncated RNAs, and because these are more abundant in Thal10 cells we repeated Actinomycin D chase but this time used S1 nuclease protection to monitor changes in full-length and 5′-truncated RNAs (Figure 1). Again, full-length PTC-hβG mRNA showed a biphasic curve similar our previous results and to PTC-hβG mRNA in non-erythroid cells [16]. Although they appeared to be more stable than full-length mRNA there were differences in the patterns seen for each of the 5′-truncated RNAs. The transcripts whose 5′ ends were closest to that of full-length mRNA disappeared at a faster rate than those whose 5′ ends were further away. Although it does not constitute proof, this is consistent with multiple endonuclease cleavage events happening over time until a limit digest is reached.

### A Quantitative Assay for Full-length and 5′-truncated β-globin RNAs

**Figure 2:**
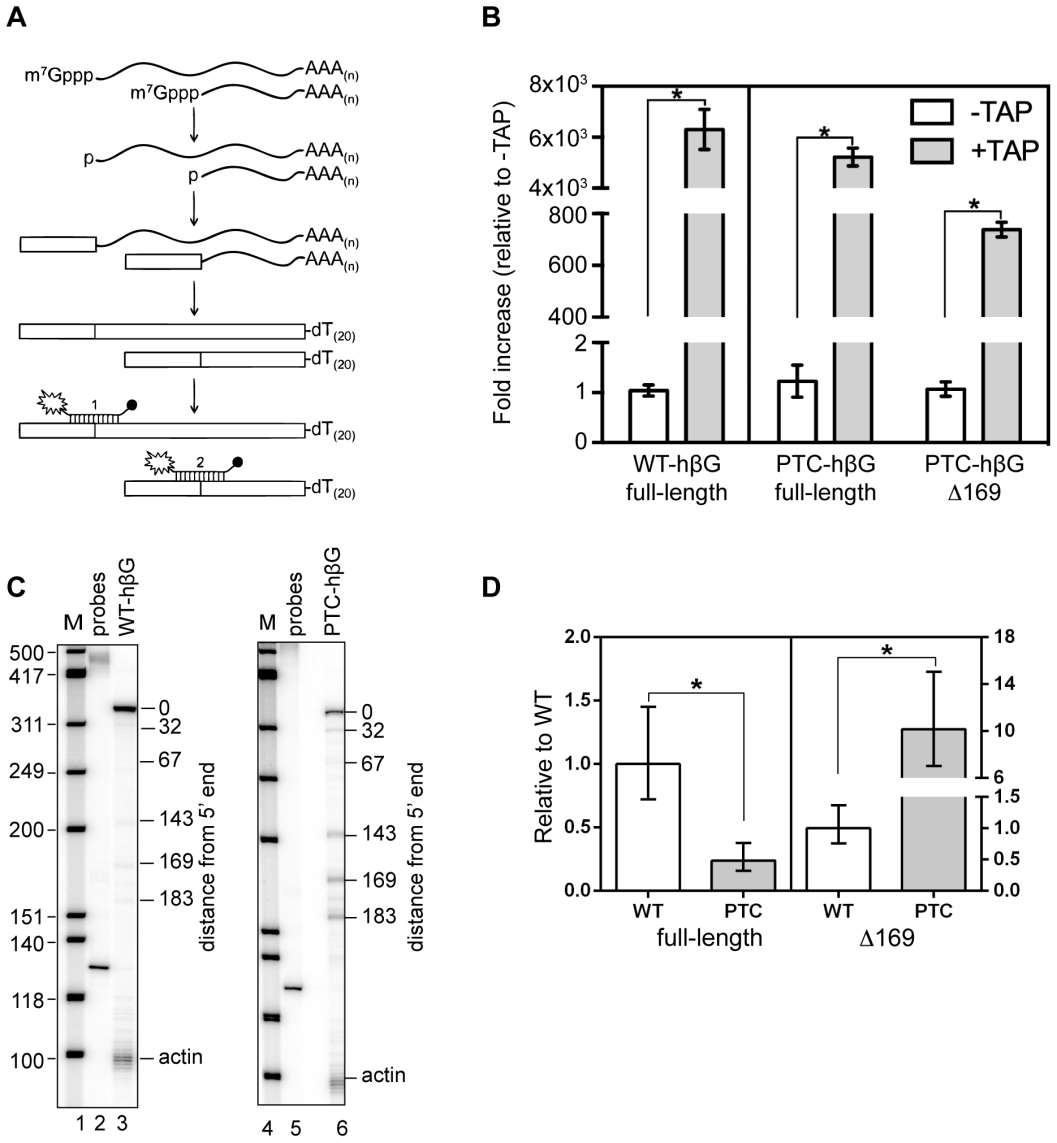
Development of a modified MBRACE assay for quantifying full-length and one of the 5′-truncated β-globin mRNAs. **A.** A schematic of the modified MBRACE assay is shown, and the locations of each of the primers are presented in Figure S1. **B.** RNA from triplicate cultures of MEL cells expressing WT-hβG or PTC-hβG mRNA was added directly to the ligation reaction with the 5′ RNA adapter (–TAP, open bars) or treated with tobacco acid pyrophosphatase (+TAP, grey bars) before ligation. Results obtained without TAP treatment were arbitrarily set to 1 and results obtained with TAP-treated RNA were normalized to the −TAP samples. The results represent the mean ± standard deviation for triplicate samples. *indicates p<0.01 by Student’s two-tailed T-test. **C.** S1 nuclease protection assay was used to demonstrate the increased accumulation of 5′-truncated RNAs in cells expressing PTC-hβG mRNA (lane 6) compared to WT-hβG mRNA (lane 3). The locations of each of the 5′-truncated RNAs with respect to the cap site are indicated to the right of each autoradiogram. **D.** The transcript beginning at position 169 nt (Δ169) was selected as a representative truncated RNA. Cytoplasmic RNA from MEL cells expressing WT- or PTC-hβG mRNA was used to demonstrate applicability of the modified MBRACE assay for quantifying changes in full-length mRNA and Δ169 RNA. In each graph the amount of RNA from each cell line was first normalized to β-actin. Shown is the mean ± standard deviation for biological triplicates, *indicates p<0.001 by two-tailed t-test.

Given the challenge presented by the prolonged presence of 5′-truncated RNAs we sought an alternative approach for studying hβG mRNA decay in erythroid cells. The first step involved the development of a method for quantifying full-length mRNA and one of the truncated transcripts, and for this we selected Molecular Beacon Rapid Amplification of cDNA Ends (MBRACE) [11] (outlined in Figure 2A). In this assay, cytoplasmic RNA is first treated with a phosphatase to prevent adapter ligation to uncapped ends. The cap is removed with tobacco acid pyrophosphatase (TAP), an RNA adapter is ligated onto the newly created 5′-monophosphate ends and the products are quantified using junction-specific molecular beacons as indicated in the figure or by qRT-PCR. Results in the left and middle panels of Figure 2B comparing untreated with TAP-treated RNA demonstrate the importance of removing the cap for MBRACE assay of full-length WT- and PTC-hβG mRNAs.

A similar approach was then developed for quantifying one of the 5′-truncated RNAs shown in the right panel of Figure 2C. 5′-RACE that was performed using TAP-treated RNA from PTC-hβG-expressing Thal10 cells and individual cloned products were sequenced to confirm that the junction with the ligated adapter matched each of the 5′ ends identified in [7] by primer extension and S1 nuclease protection. The transcript with 5′ end at position 169 (Δ169) was then selected for assay development. Our discovery of cytoplasmic capping [17] was the result of following an earlier report describing the 5′ ends of the truncated RNAs as having a cap or cap-like structure [18]. The presence of a cap on 5′-truncated RNAs is evident in the right panel of Figure 2B, where TAP treatment of RNA from PTC-hβG-expressing cells resulted in an almost 800-fold increase in signal for Δ169 RNA. Finally, the applicability of MBRACE assay for quantifying changes in full-length and Δ169 RNA was confirmed by the comparable results obtained by MBRACE assay (Figure 2D) and S1 nuclease protection (Figure 2C) performed with RNA from MEL cells expressing WT- and PTC-hβG mRNA.

### NMD is Responsible for the Increase in 5′ Truncated RNAs from PTC-hβG mRNA

**Figure 3:**
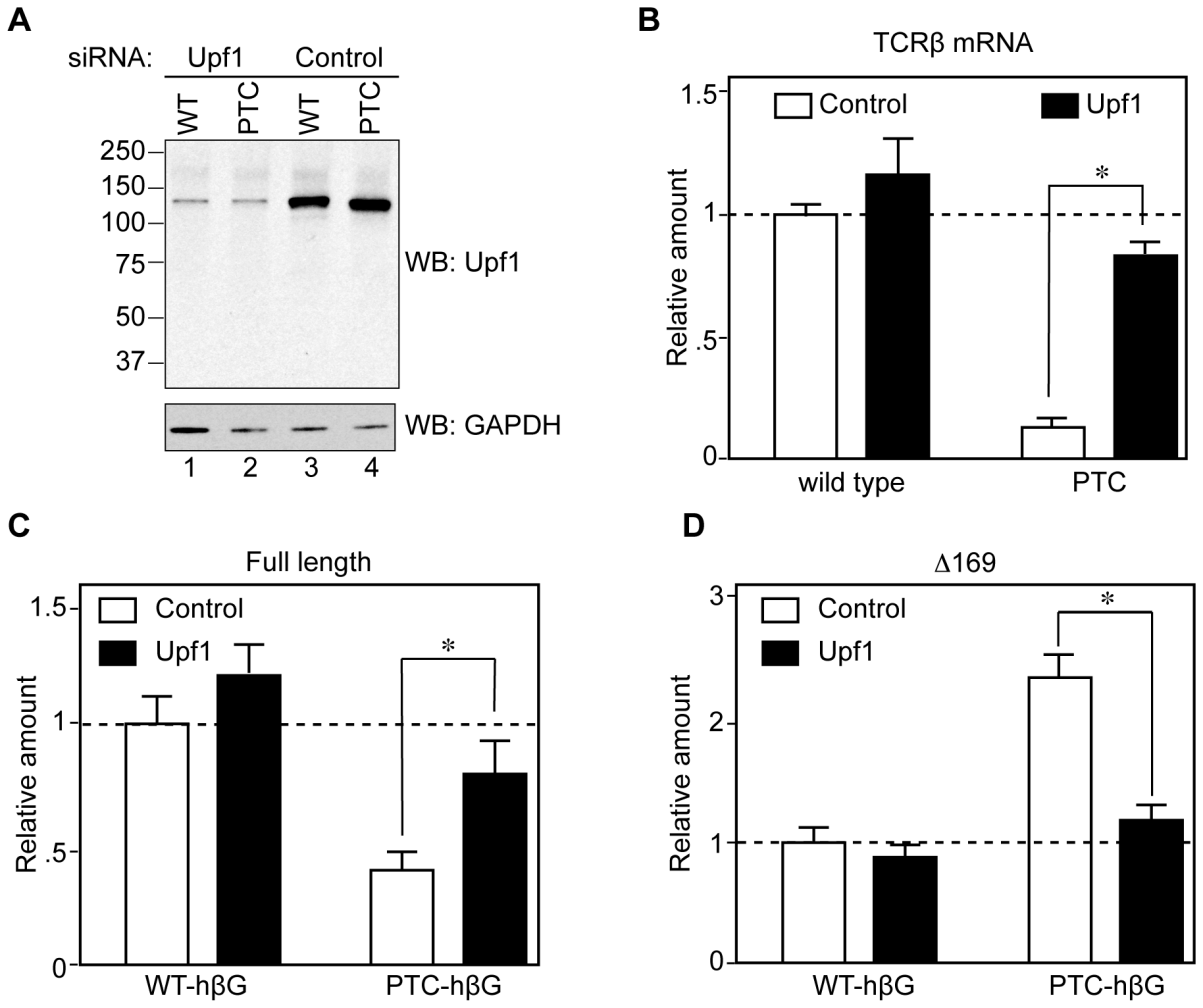
Evidence that NMD is responsible for the accumulation of Δ169 RNA from PTC−hβG mRNA. Tet-inducible K562 cells in antibiotic-free medium were electroporated with plasmids expressing constitutive wild type or nonsense-containing TCRβ genes, or each of the inducible β-globin genes, and a GFP control. Sixteen hr later they were transfected with Accell SmartPool→ Upf1 or control siRNAs, cultured for an additional 48 hr, then induced with doxycycline for 6 hr. A. Cytoplasmic extracts from WT- and PTC-hβG expressing cells were assayed by Western blotting for efficiency of Upf1 knockdown. **B.** The effectiveness of Upf1 knockdown in inhibiting NMD was determined by qRT-PCR analysis of changes in WT- versus PTC-TCRβ mRNA. **C.** The modified MBRACE assay was used to quantify the impact of Upf1 knockdown on full-length WT- and PTC-hβG mRNA. **D.** Modified MBRACE assay was used to monitor the impact of Upf1 knockdown on the production of Δ169 RNA. The results represent the mean ± standard deviation of triplicate cultures, *indicates p<0.05 by two-tailed Student’s T-test.

Although K562 cells are an erythroid cell line they do not express hβG mRNA. We developed a line of tetracycline inducible K562 cells to circumvent complications resulting from the prolonged cytoplasmic lifetime of 5′-truncated RNAs. In each of the following experiments these cells were electroporated with plasmids expressing tetracycline inducible full-length WT- and PTC-hβG genes, and changes in full-length and Δ169 RNA were quantified by MBRACE assay after inducing their transcription with doxycycline. In the experiment in Figure 3 electroporated K562 cells were treated with Accell® control or Upf1 siRNAs before inducing WT- or PTC-hβG mRNA. This reduced Upf1 to 10% of control (Figure 3A), and the stabilization of nonsense-containing TCRβ mRNA (Figure 3B) demonstrated its effectiveness in inactivating NMD. Knocking down Upf1 had little impact on WT-hβG mRNA or the amount of Δ169 RNA in cells expressing WT-hβG mRNA; however, the stabilization of full-length PTC-hβG mRNA and reduction in the level of Δ169 RNA to that observed with WT-hβG (Figure 3C and D) confirm that NMD is responsible for the increased appearance of 5′-truncated transcripts from PTC-hβG mRNA.

### The 5′ Truncated RNAs are Metastable Decay Intermediates

**Figure 4:**
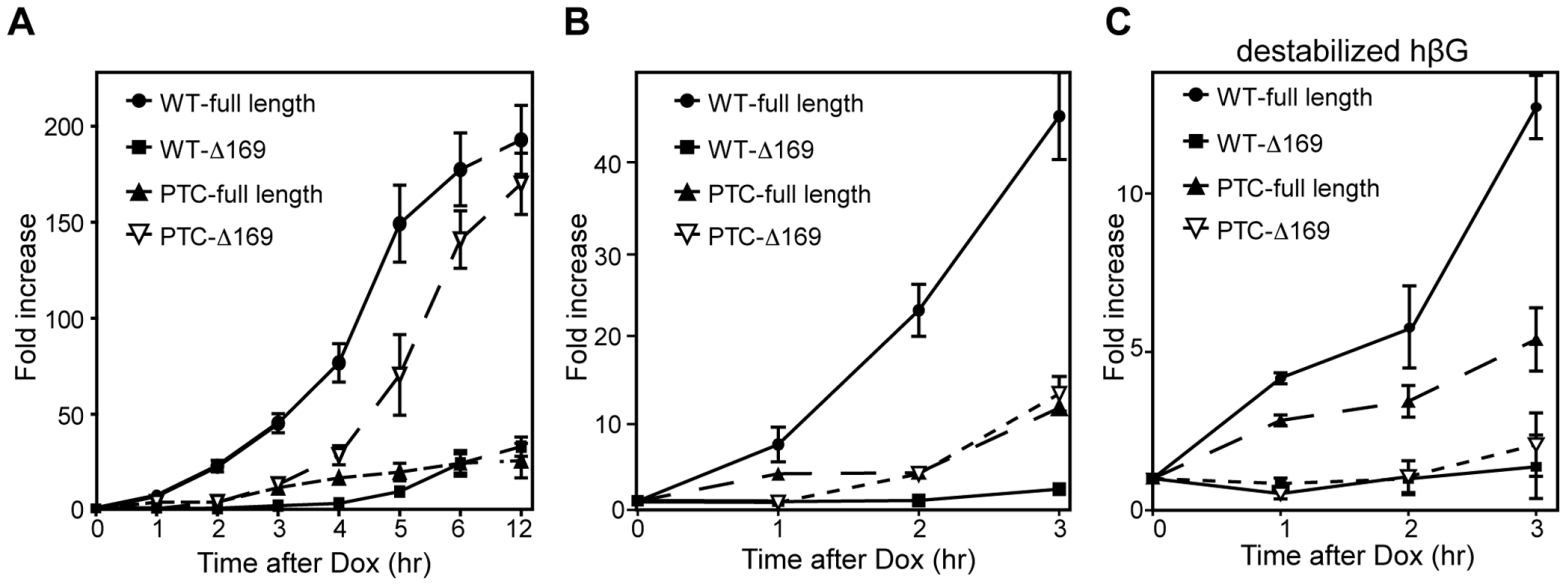
Evidence that 5′-truncated RNAs are decay intermediates. **A.** Doxycycline was added at time 0 to tet-inducible K562 cells that were electroporated 16 hr earlier with plasmids bearing inducible WT- or PTC-hβG genes and a GFP control. Cytoplasmic RNA isolated at intervals over 12 hr of induction was analyzed by modified MBRACE assay for changes in full-length and Δ169 forms of hβG mRNA. **B.** The first 3 hr of induction is shown enlarged. **C.** The experiment was repeated except that the plasmids that were electroporated into tet-inducible K562 cells expressed a destabilized form of β-globin mRNA (H124 mutation) as a result of disruption of the 3′-UTR nucleolin binding site [12]. Each point represents the mean ± standard deviation for triplicate determinations.

The prolonged cytoplasmic residence of 5′-truncated RNAs after Actinomycin D (Figure 1) ruled out the use of transcription inhibitors in determining whether these are intermediates of PTC-hβG decay. Instead we monitored the appearance of both forms of hβG mRNA over time after inducing transcription of their respective WT- and PTC-hβG genes (Figure 4). If the truncated RNAs are decay intermediates the expectation is their appearance in the cytoplasm should follow that of full-length mRNA. Changes in each transcript over 12 hr after induction are shown in Figure 4A, and the first 3 hr after induction, where initial differences are more evident, are shown enlarged in (Figure 4B). The appearance of Δ169 RNA after full-length WT- and PTC-hβG mRNA is consistent with assignment of the shortened RNAs as intermediates in the decay process.

To obtain more definitive evidence of this we looked for a way to accentuate the differences in time of appearance of full-length and Δ169 RNA that was compatible with the inducible cell system. To address this we took advantage of the fact that given the same degree of induction an mRNA that turns over rapidly will reach a new steady state sooner than one with a slower rate of decay [19]. The prolonged stability of hβG mRNA (and possibly the 5′ truncated transcripts) is due in part from the binding of nucleolin to a site in the 3′-UTR [12]. In the experiment in Figure 4C the time course appearance of the different RNAs was repeated using WT- and PTC-βG genes with inactivated nucleolin binding sites (H124, [12]. As anticipated, this reduced the degree to which of each mRNA accumulated; however, it provided clear and convincing evidence of differences in the time of appearance of full-length and Δ169 RNAs. Taken together with results in [7], [8] these data confirm that the 5′-truncated RNAs are endonuclease-generated decay intermediates.

### SMG6 is the Endonuclease that Generates 5′-truncated forms of PTC-hβG mRNA

**Figure 5:**
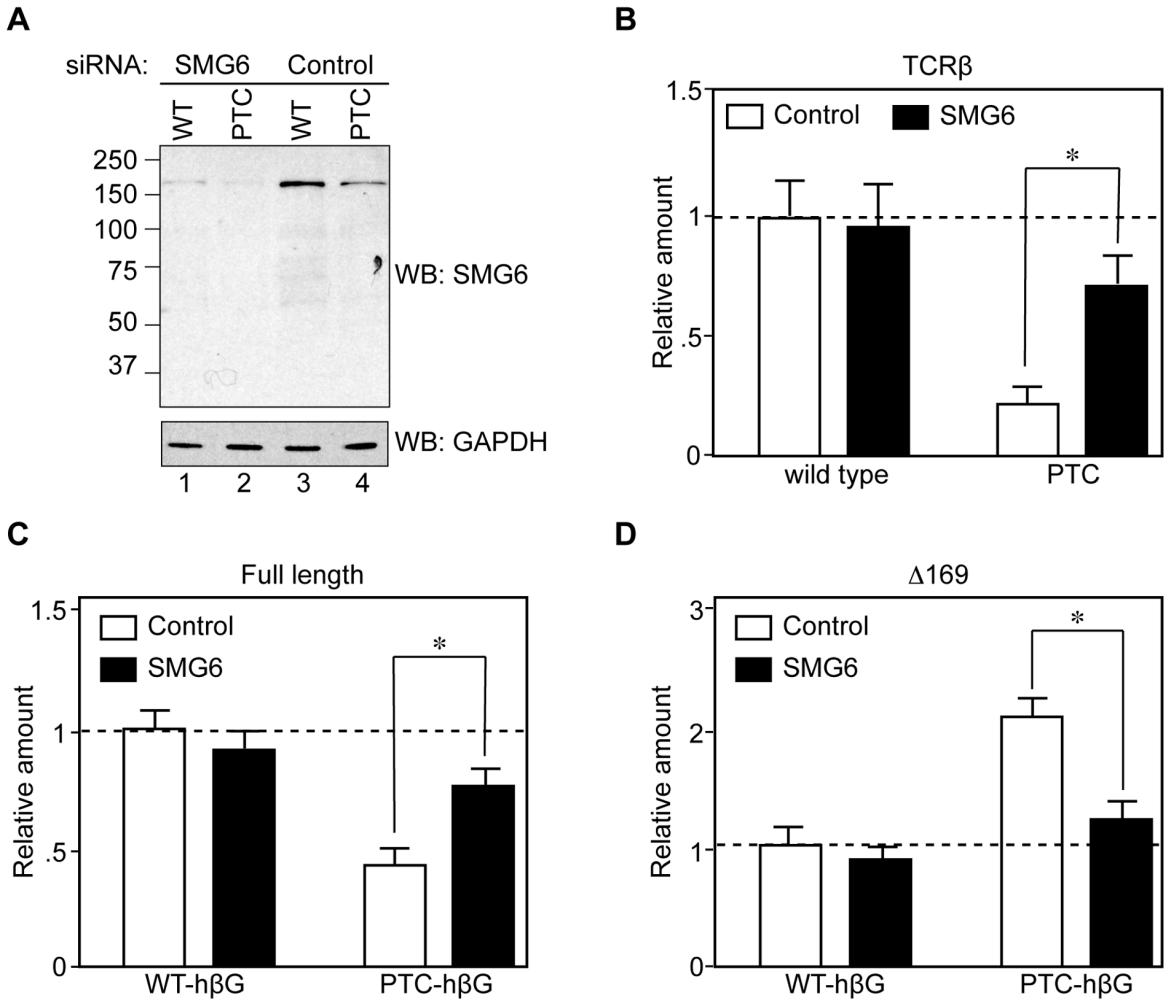
SMG6 knockdown increases full-length PTC-hβG mRNA and decreases Δ169 RNA. Tet-inducible K562 cells in antibiotic-free medium were electroporated as in Figure 3, with plasmids expressing constitutive WT- or PTC-TCRβ genes, or each of the inducible hβG genes, and a GFP control. Sixteen hr later they were transfected with Accell SmartPool→ SMG6 or control siRNAs, cultured for an additional 48 hr, then induced with doxycycline for 6 hr. **A.** Cytoplasmic extracts from cells expressing each form of β-globin mRNA were assayed by Western blotting for efficiency of SMG6 knockdown. **B.** The effectiveness of SMG6 knockdown in inhibiting NMD was determined by qRT-PCR analysis of changes in WT- versus PTC-TCRβ mRNA. **C.** The modified MBRACE assay was used to quantify the impact of SMG6 knockdown on full-length WT- and PTC-hβG mRNA. **D.** Modified MBRACE assay was used to monitor the impact of SMG6 knockdown on the production of Δ169 RNA. The results represent the mean ± standard deviation of triplicate cultures, *indicates p<0.05 by two-tailed Student’s t-test.

We next sought to determine the identity of the endonuclease responsible for generating the 5′-truncated decay intermediates. Our previous work suggested this was PMR1 or a PMR1-like endonuclease [7], [8]. However this was ruled out by the lack of any impact on WT- or PTC-hβG mRNA of inhibiting PMR1 activation or overexpressing human PMR1 (Figure S2). SMG6 is a PIN-domain containing endonuclease that participates in the degradation of nonsense-containing mRNAs [9], [10]. It lacks sequence selectivity, and specificity for nonsense-containing mRNAs results from its binding to the exon junction at the same site as Upf3b [20]. In the experiment in Figure 5 K562 cells electroporated with plasmids expressing WT- and PTC-hβG mRNA were knocked down for SMG6 before adding doxycycline to induce WT- and PTC-hβG mRNA. Knockdown depleted ∼90% of SMG6 (Figure 5A) and this had the anticipated effect of stabilizing PTC-containing TCRβ mRNA (Figure 5B). SMG6 knockdown had no impact on WT full-length or Δ169 RNA, increased full-length PTC-hβG mRNA and returned the amount Δ169 RNA to the level generated from WT-hβG mRNA.

**Figure 6:**
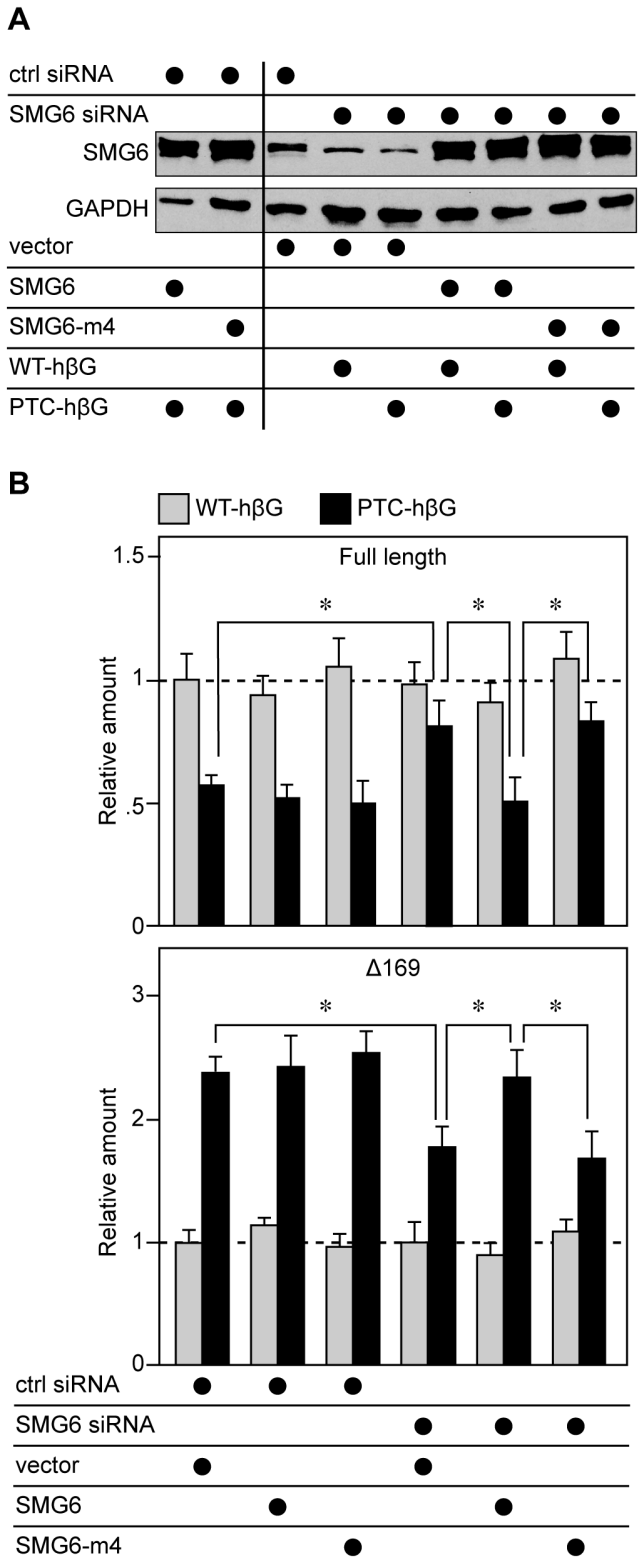
Complementation identifies SMG6 is the endonuclease responsible for generating Δ169 RNA from PTC-hβG mRNA. Tet-inducible K562 cells in antibiotic-free medium were electroporated as in Figure 3, with control or SMG6 siRNAs. Twenty-four hr later they were electroporated again with the same siRNAs plus empty vector or plasmids expressing siRNA resistant SMG6 or a catalytically-inactive form of SMG6 (SMG6-m4) and plasmids expressing each of the inducible hβG genes. Doxycycline was added 16 hr later to induce each of the hβG genes. **A.** Cytoplasmic extracts from triplicate cultures were pooled and analyzed by Western blotting with anti-SMG6 antibody or with antibody to GAPDH. **B.** Cytoplasmic RNA from individual cultures was analyzed by modified MBRACE assay for full-length (upper panel) and Δ169 (lower panel) forms of hβG mRNA. The results represent the mean ± standard deviation of triplicate cultures, *indicates p<0.05 by two-tailed Student’s t-test.

Although these data are consistent with SMG6 generating Δ169 RNA it was formally possible that the observed effects were secondary to changes in Upf1 phosphorylation [21]. This was first addressed by a complementation experiment similar to that performed in [10]. In the experiment in Figure 6 tetracycline-inducible K562 cells were electroporated with control or SMG6 siRNAs, then a second time with the same siRNAs together with empty vector or plasmids expressing siRNA-resistant SMG6 or an inactive form of SMG6 with the 3 active site aspartic acid residues changed to asparagine (SMG6-m4). The second round of transfection included tetracycline-inducible plasmids expressing WT- and PTC-hβG mRNA, and cells recovered 6 hr after induction were analyzed by Western blotting for changes in SMG6 and by modified MBRACE assay.

SMG6 knockdown reduced levels of the endogenous protein to ∼30% of control (Figure 6A, compare lane 3 with lanes 4 and 5), and in each transfectant siRNA-resistant SMG6 was overexpressed compared to endogenous protein (Figure 6A, compare lanes 1,2,6–9 with lane 3). The similar expression of each form of recombinant SMG6 confirmed that each of these is resistant to SMG6 siRNA and therefore capable of complementing the impact of SMG6 knockdown on PTC-hβG NMD. Results in Figure 6B show that neither SMG6 knockdown nor its complementation with exogenous SMG6 had any impact on wild-type full-length or Δ169 RNA (grey bars). As in Figure 5, SMG6 knockdown increased the amount of full-length PTC-hβG mRNA and decreased the amount of Δ169 RNA (SMG6 siRNA+vector). This was reversed by co-expression of siRNA-resistant SMG6, but not by co-expression of catalytically-inactive SMG6-m4, thus confirming the identity of SMG6 as the enzyme that is responsible for generating stable decay products from PTC-hβG mRNA.

### Altering SMG6 has no Impact on the Phosphorylation State of Upf1

**Figure 7:**
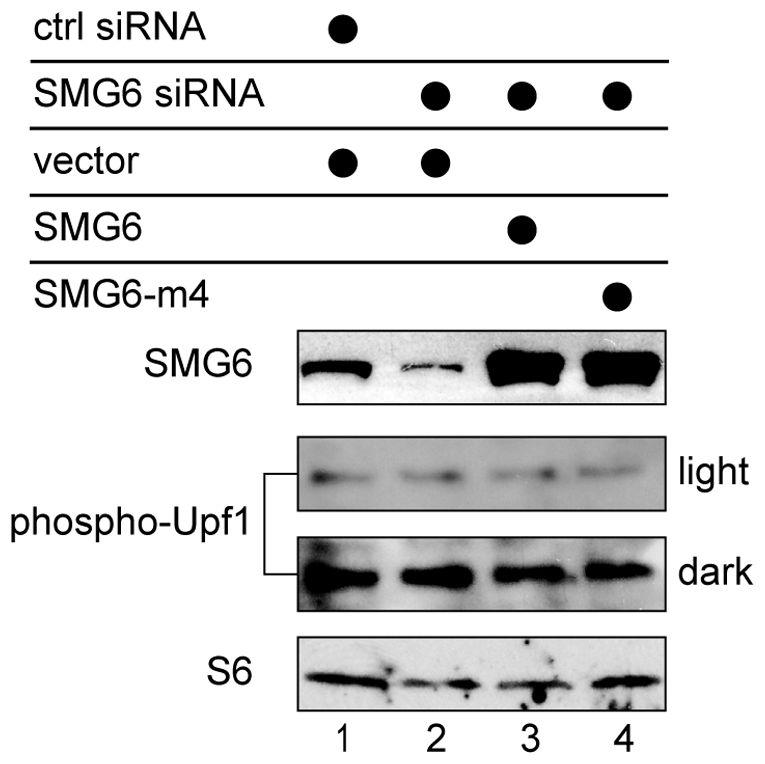
Upf1 phosphorylation is unaffected by SMG6 knockdown or overexpression of inactive SMG6. Tet-inducible K562 cells were electroporated as in Figure 6 with control or SMG6 siRNAs and empty vector, or plasmids expressing SMG6 or inactive SMG6-m4. Cytoplasmic extracts recovered 16 hr after the second round of electroporation were analyzed by Western blotting with antibodies to SMG6 (top panel) or to phospho-Upf1 (middle panels). Both light and dark exposures are shown. Ribosomal protein S6 was used as a loading control (bottom panel).

In Okada-Katsuhata et al. [21] NMD was inhibited by SMG6 knockdown or by overexpression of an inactive form of SMG6. SMG6 functions in the dephosphorylation of Upf1, and in each case this was reported to result from interference with the cycling of Upf1 between phosphorylation states. The absence of an inhibitory effect of SMG6m4 on PTC-hβG mRNA decay in Figure 6B argued against this. Nevertheless, to be certain that the results in Figs. 5 and 6 indeed demonstrate a direct role for SMG6 endonuclease activity in generating stable decay intermediates we looked at the impact of each of these changes on the phosphorylation state of Upf1. In the experiment in Figure 7 K562 cells were electroporated as in Figure 6 with control or SMG6 siRNAs, and with empty vector or with plasmids expressing SMG6 or SMG6-m4. Western blotting with anti-SMG6 antibody (top panel) confirmed the effectiveness of each of these steps in reducing or increasing each form of SMG6. Importantly, Western blotting with an anti-phosphoUpf1 antibody showed that none of these treatments had any impact on the phosphorylation state of Upf1.

## Discussion

To the best of our knowledge the 5′-truncated forms of PTC-hβG mRNA examined here are the only example of metastable products of nonsense-mediated mRNA decay. They were first identified in erythroid cells of mice expressing a nonsense-containing transgene [6], and to date have only been detected in erythroid cells. Like the parent mRNA the 5′ ends of these RNAs are capped [17], [18] and they have an intact 3′-poly(A) tail [7]. S1 nuclease protection assays performed on cytoplasmic RNA recovered over 8 hr of actinomycin D treatment showed full-length PTC-hβG mRNA decayed with biphasic kinetics (Figure 1) that resemble the decay of PTC-hβG mRNA in non-erythroid cells [16]. The decay curves in Figure 1B indicated that the 5′-truncated RNAs disappear more slowly than full-length PTC-hβG mRNA. However, the transcripts with 5′ ends closer to the cap appear to decay more quickly than transcripts whose 5′ ends are further from the cap, the result one might expect for RNA undergoing multiple cleavage events until reaching some limit digest. The complexity of this precluded the use transcriptional inhibition to study the relationship of the 5′-truncated transcripts to PTC-hβG mRNA decay. This was instead addressed through the use of an inducible erythroid cell line and a modification of the MBRACE assay for quantifying changes in full-length mRNA and one of the truncated RNAs (Δ169). In the course of qualifying this assay we reaffirmed results in [17] showing the 5′-truncated RNAs are capped (Figure 2B).

Nonsense-containing mRNAs have been reported to undergo deadenylation, decapping, 5′-exonucleolytic decay, 3′-exonucleolytic decay and endonucleolytic decay [2], [22]. The rules by which any given mRNA selected for a particular degradation pathway have not been determined. Results presented here confirm that NMD is responsible for the decay of full-length PTC-hβG mRNA and identify the 5′-truncated RNAs as decay intermediates. Three different experiments demonstrated that SMG6 is the endonuclease that generates these intermediates. The first (Figure 5) looked at the impact of SMG6 knockdown alone, and the most straightforward interpretation of these data identifies SMG6 is the enzyme that cleaves PTC-hβG mRNA to generate the stable Δ169 intermediate. However, SMG6 has been reported to function in the dephosphorylation of Upf1, and its knock down in HeLa cells was reported to inhibit NMD secondary to the accumulation of phospho-Upf1 [21]. To address this we performed a complementation experiment in which we knocked down SMG6 and expressed siRNA-resistant forms of SMG6 or SMG6 with the 3 aspartic acid residues in the PIN domain catalytic core changed to asparagine (SMG6-m4). Results in Figure 6 show that expression of siRNA resistant SMG6 reversed the effect of SMG6 knockdown on the generation of Δ169 RNA from PTC-hβG mRNA, but expression of SMG6-m4 did not, thus supporting the identification of SMG6 as the responsible endonuclease.

Results in Figure 6 also brought to light a difference between our results and those in [21] regarding the impact of overexpressing inactive forms of SMG6. In that study NMD was inhibited by overexpression of SMG6 with a single aspartate-to-alanine PIN domain mutation (D1251A). We saw no evidence for inhibition of NMD with overexpression of SMG6-m4 in K562 cells. In Figure 6 each form of SMG6 was clearly overexpressed (Figure 6A); however, as evident in the first 3 datasets in Figure 6B neither of these had any impact on full-length or Δ169 RNA. We do not know why these results differ from those in [21], but this may be due to differences in the form of inactive SMG6 used in our study, or to the fact that our experiments examined hβG mRNA in its native cell context.

It remained formally possible that SMG6 knockdown still resulted in the accumulation of phosho-Upf1 and expression of SMG6-m4 just helped to keep Upf1 in the phosphorylated state. This was ruled out by the results of Figure 7, where we saw no evidence for changes in the phosphorylation state of Upf1 regardless of whether SMG6 was knocked down or an inactive form of SMG6 was expressed in these cells. Together with the preceding experiments these data provide proof that metastable decay intermediates are generated by SMG6 cleavage of PTC-hβG mRNA.

Finally, K562 cells do not natively express β-globin mRNA but they do express detectable levels of δ-globin mRNA [23]. In the course of this work we identified a form of δ-globin mRNA whose 5′ end matched that of Δ169 RNA (Figure S1). This raised a question that was not answered by this study; to wit, why are the same 5′-truncated RNAs also detectable in cells expressing WT-hβG mRNA? These were seen in [7] and [8], and again here by both S1 nuclease protection and by the modified MBRACE assay (Figure 2). Although the endonuclease responsible for generating these fragments has yet to be identified, the fact that both WT- and PTC-hβG mRNA are cleaved at the same sites suggests features of the mRNP play a major role in determining the location of endonuclease cleavage sites.

## Supporting Information

Figure S1 **Sequence alignment of human beta- and delta-globin mRNA and locations of MBRACE primers.** Human beta-globin (HBB) and delta-globin (HBD) mRNAs are shown aligned. The yellow highlights identify the locations of primers used to quantify full-length mRNA and the green highlights identify the locations of primers used to quantify D169 RNA. Note that the sequence at the 5′ D169 primer binding site is identical for beta- and delta-globin mRNA.
(TIF)

Figure S2 **Impact of changes in PMR1 on full-length and Δ169 hβG mRNA.** **A** and **B.** Tet-inducible K562 cells electroporated with inducible WT- and PTC-hβG genes were treated with DMSO (vehicle), PP3 (an inactive analog of PP2), or PP2 c-Src inhibitor to inactivate PMR1 targeting to polysomes [24], [25]. Cytoplasmic RNA recovered 6 hr after induction was assayed by modified MBRACE for changes in full-length (**A**) and Δ169 RNA (**B**). **C** and **D**. Tet-inducible K562 cells were electroporated with WT- and PTC-hβG expressing plasmids together with plasmids expressing GFP, active hPMR1 or inactive hPMR1. Cytoplasmic RNA recovered 6 hr after induction was analyzed by modified MBRACE for changes in full-length (**C**) and Δ169 RNA (**D**). The results represent the mean ± standard deviation of triplicate cultures, *indicates p<0.05 by two-tailed Student’s t-test.
(TIF)

Table S1 Oligonucleotides and primers.
(XLSX)

## References

[1] Karginov FV, Cheloufi S, Chong MMW, Start A, Smith AD, et al (2010) Diverse endonucleolytic cleavage sites in the mammalian transcriptome depend on microRNAs, Drosha and additional nucleases. Mol Cell 38: 781–788. 20620951 10.1016/j.molcel.2010.06.001 PMC2914474

[2] Schoenberg DR, Maquat LE (2012) Regulation of cytoplasmic mRNA decay. Nat Rev Genet 13: 246–259. 22392217 10.1038/nrg3160 PMC3351101

[3] Schoenberg DR (2011) Mechanisms of endonuclease-mediated mRNA decay. Wiley Interdisc Rev: RNA 2: 582–600. 10.1002/wrna.78 PMC3347869 21957046

[4] Gatfield D, Izaurralde E (2004) Nonsense-mediated messenger RNA decay is initiated by endonucleolytic cleavage in *Drosophila* . Nature 429: 575–578. 15175755 10.1038/nature02559

[5] Hanson MN, Schoenberg DR (2001) Identification of *in vivo* mRNA decay intermediates corresponding to sites of in vitro cleavage by polysomal ribonuclease 1. J Biol Chem 276: 12331–12337. 11152474 10.1074/jbc.M010483200 PMC2262841

[6] Lim S, Mullins JJ, Chen CM, Gross KW, Maquat LE (1989) Novel metabolism of several β°-thalassemic β-globin mRNAs in the erythroid tissues of transgenic mice. EMBO J 8: 2613–2619. 2573525 10.1002/j.1460-2075.1989.tb08401.x PMC401267

[7] Stevens A, Wang Y, Bremer K, Zhang J, Hoepfner R, et al (2002) Beta-globin mRNA decay in erythroid cells: UG site-preferred endonucleolytic cleavage that is augmented by a premature termination codon. Proc Natl Acad Sci U S A 99: 12741–12746. 12242335 10.1073/pnas.192442399 PMC130530

[8] Bremer KA, Stevens A, Schoenberg DR (2003) An endonuclease activity similar to *Xenopus* PMR1 catalyzes the degradation of normal and nonsense-containing human beta-globin mRNA in erythroid cells. RNA 9: 1157–1167. 12923263 10.1261/rna.5720303 PMC1370479

[9] Huntzinger E, Kashima I, Fauser M, Sauliere J, Izaurralde E (2008) SMG6 is the catalytic endonuclease that cleaves mRNAs containing nonsense codons in metazoan. RNA 14: 2609–2617. 18974281 10.1261/rna.1386208 PMC2590965

[10] Eberle AB, Lykke-Andersen S, Muhlemann O, Jensen TH (2009) SMG6 promotes endonucleolytic cleavage of nonsense mRNA in human cells. Nat Struct Mol Biol 16: 49–55. 19060897 10.1038/nsmb.1530

[11] Lasham A, Herbert M, Coppieters ’t Wallant N, Patel R, Feng S, et al (2010) A rapid and sensitive method to detect siRNA-mediated mRNA cleavage in vivo using 5′ RACE and a molecular beacon probe. Nucleic Acids Res 38: e19. 19942683 10.1093/nar/gkp1076 PMC2817477

[12] Jiang Y, Xu XS, Russell JE (2006) A nucleolin-binding 3′ untranslated region element stabilizes beta-globin mRNA *in vivo* . Mol Cell Biol 26: 2419–2429. 16508016 10.1128/MCB.26.6.2419-2429.2006 PMC1430272

[13] Wang J, Vock VM, Li S, Olivas OR, Wilkinson MF (2002) A quality control pathway that down-regulates aberrant T-cell receptor (TCR) transcripts by a mechanism requiring UPF2 and translation. J Biol Chem 277: 18489–18493. 11889124 10.1074/jbc.M111781200

[14] Otsuka Y, Schoenberg DR (2008) Approaches for studying PMR1 endonuclease-mediated mRNA decay. Methods Enzymol 448: 241–263. 19111180 10.1016/S0076-6879(08)02613-X PMC2734451

[15] Livak KJ, Schmittgen TD (2001) Analysis of relative gene expression data using real-time quantitative PCR and the 2(-Delta Delta C(T)) Method. Methods 25: 402–408. 11846609 10.1006/meth.2001.1262

[16] Trcek T, Sato H, Singer RH, Maquat LE (2013) Temporal and spatial characterization of nonsense-mediated mRNA decay. Genes Dev 27: 541–551. 23431032 10.1101/gad.209635.112 PMC3605467

[17] Otsuka Y, Kedersha NL, Schoenberg DR (2009) Identification of a cytoplasmic complex that adds a cap onto 5′-monophosphate RNA. Mol Cell Biol 29: 2155–2167. 19223470 10.1128/MCB.01325-08 PMC2663312

[18] Lim SK, Maquat LE (1992) Human beta-globin mRNAs that harbor a nonsense codon are degraded in murine erythroid tissues to intermediates lacking regions of exon I or exons I and II that have a cap-like structure at the 5′ termini. EMBO J 11: 3271–3278. 1324170 10.1002/j.1460-2075.1992.tb05405.x PMC556861

[19] Ross J (1995) mRNA stability in mammalian cells. Microbiol Rev 59: 423–450. 7565413 10.1128/mr.59.3.423-450.1995 PMC239368

[20] Kashima I, Jonas S, Jayachandran U, Buchwald G, Conti E, et al (2010) SMG6 interacts with the exon junction complex via two conserved EJC-binding motifs (EBMs) required for nonsense-mediated mRNA decay. Genes Dev 24: 2440–2450. 20930030 10.1101/gad.604610 PMC2964754

[21] Okada-Katsuhata Y, Yamashita A, Kutsuzawa K, Izumi N, Hirahara F, et al (2012) N- and C-terminal Upf1 phosphorylations create binding platforms for SMG-6 and SMG-5:SMG-7 during NMD. Nucleic Acids Res 40: 1251–1266. 21965535 10.1093/nar/gkr791 PMC3273798

[22] Kervestin S, Jacobson A (2012) NMD: a multifaceted response to premature translational termination. Nat Rev Mol Cell Biol 13: 700–712. 23072888 10.1038/nrm3454 PMC3970730

[23] Poddie D, Marongiu MF, Ferrari SC, Porcu S, Ristaldi MS (2003) delta-Globin gene structure and expression in the K562 cell line. Hemoglobin 27: 219–228. 14649312 10.1081/hem-120026046

[24] Yang F, Peng Y, Schoenberg DR (2004) Endonuclease-mediated mRNA decay requires tyrosine phosphorylation of polysomal ribonuclease 1 (PMR1) for the targeting and degradation of polyribosome bound substrate mRNA. J Biol Chem 279: 48993–49002. 15375158 10.1074/jbc.M409776200 PMC1578673

[25] Peng Y, Schoenberg DR (2007) c-Src activates endonuclease-mediated mRNA decay. Mol Cell 25: 779–787. 17349962 10.1016/j.molcel.2007.01.026 PMC1861838

