# Supplementary figures and images for: SMG6 Cleavage Generates Metastable Decay Intermediates from Nonsense-Containing β-Globin mRNA
Source: PLoS One. 2013 Sep 25;8(9):e74791. doi: 10.1371/journal.pone.0074791 (PMC3783490; doi:10.1371/journal.pone.0074791)

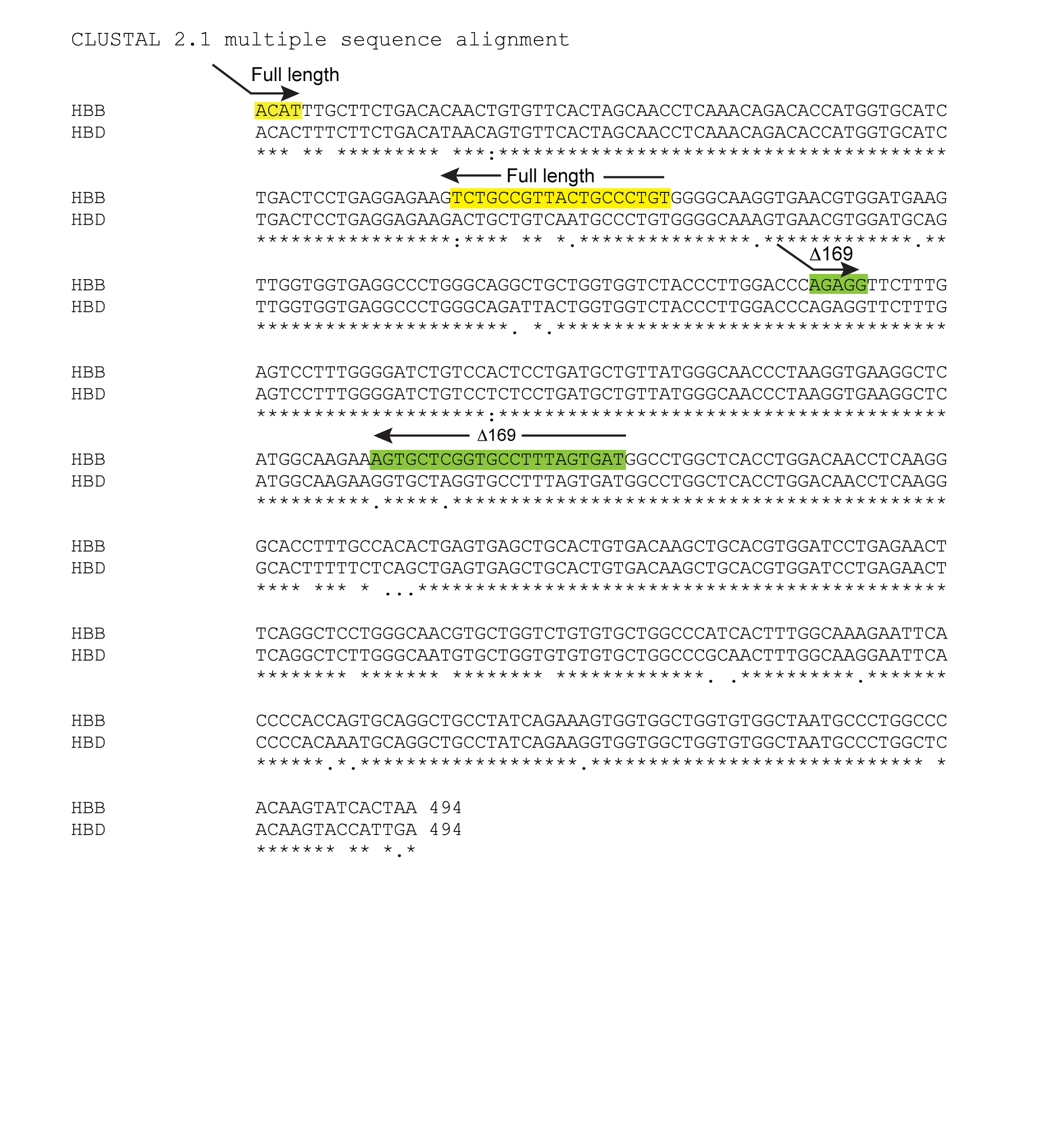

Supplement: Figure S1 — Sequence alignment of human beta- and delta-globin mRNA and locations of MBRACE primers. Human beta-globin (HBB) and delta-globin (HBD) mRNAs are shown aligned. The yellow highlights identify the locations of primers used to quantify full-length mRNA and the green highlights identify the locations of primers used to quantify D169 RNA. Note that the sequence at the 5′ D169 primer binding site is identical for beta- and delta-globin mRNA. (TIF) [file pone.0074791.s001.tif]

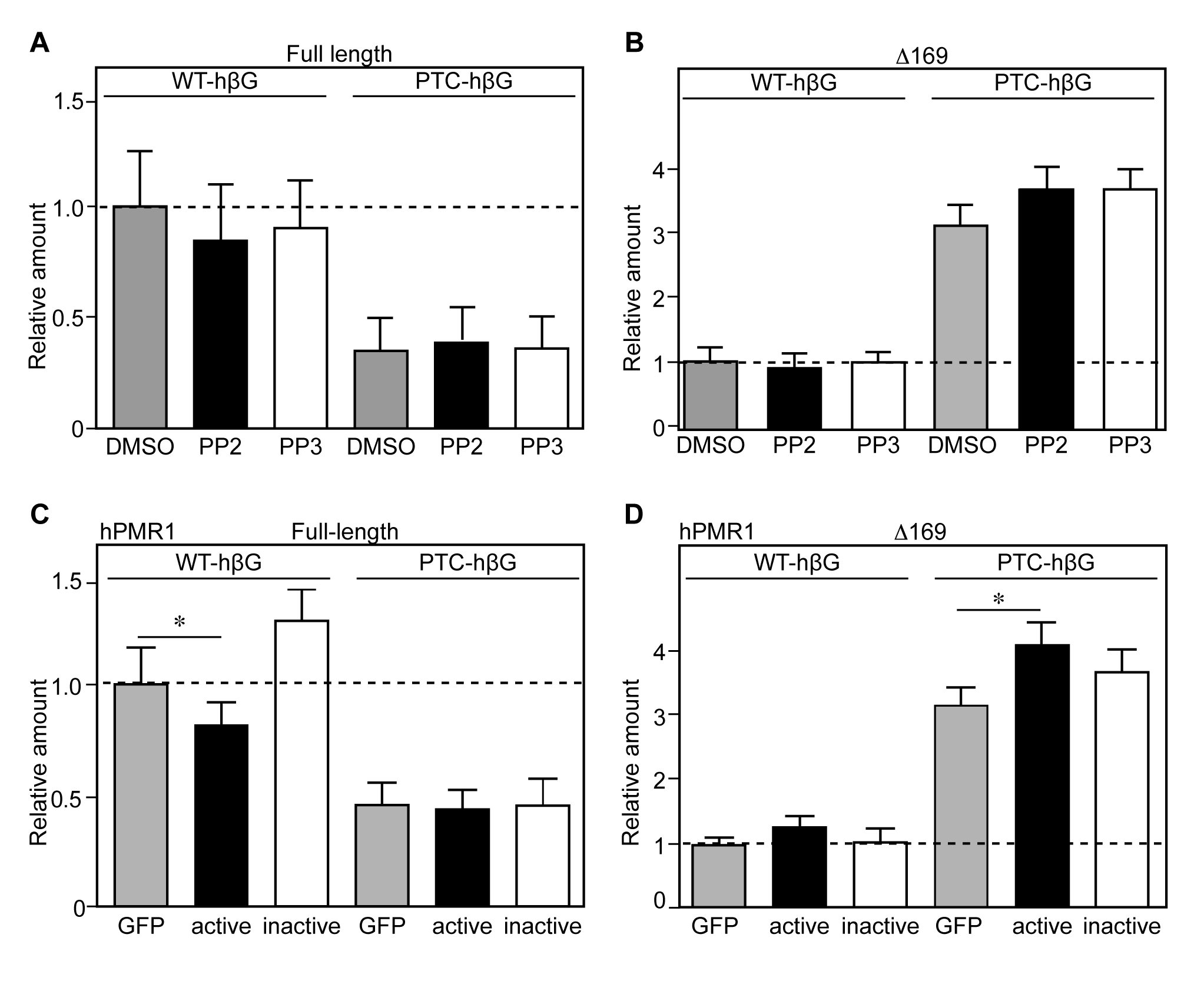

Supplement: Figure S2 — Impact of changes in PMR1 on full-length and Δ169 hβG mRNA. A and B. Tet-inducible K562 cells electroporated with inducible WT- and PTC-hβG genes were treated with DMSO (vehicle), PP3 (an inactive analog of PP2), or PP2 c-Src inhibitor to inactivate PMR1 targeting to polysomes [24], [25]. Cytoplasmic RNA recovered 6 hr after induction was assayed by modified MBRACE for changes in full-length (A) and Δ169 RNA (B). C and D. Tet-inducible K562 cells were electroporated with WT- and PTC-hβG expressing plasmids together with plasmids expressing GFP, active hPMR1 or inactive hPMR1. Cytoplasmic RNA recovered 6 hr after induction was analyzed by modified MBRACE for changes in full-length (C) and Δ169 RNA (D). The results represent the mean ± standard deviation of triplicate cultures, *indicates p<0.05 by two-tailed Student’s t-test. (TIF) [file pone.0074791.s002.tif]
